# Comprehensive metabolomic analysis of first-trimester serum identifies biomarkers of early-onset hypertensive disorder of pregnancy

**DOI:** 10.1038/s41598-020-70974-3

**Published:** 2020-08-17

**Authors:** Hyo Kyozuka, Toma Fukuda, Tsuyoshi Murata, Yuta Endo, Aya Kanno, Shun Yasuda, Akiko Yamaguchi, Miho Ono, Akiko Sato, Koichi Hashimoto, Keiya Fujimori

**Affiliations:** 1 Department of Obstetrics and Gynecology, School of Medicine, Fukushima Medical University, 1 Hikarigaoka, Fukushima, 960-1295 Japan; 2 Fukushima Regional Center for the Japan Environmental and Children’s Study, 1, Hikarigaoka, Fukushima, 960-1295 Japan

**Keywords:** Biochemistry, Biomarkers

## Abstract

Hypertensive disorders of pregnancy (HDP) lead to the death of approximately 30,000 women annually, and the identification of biomarkers to predict their onset before symptom occurrence is crucial. Here, we aimed to identify the first-trimester maternal serum biomarkers for predicting early-onset HDP via a comprehensive metabolomic analysis. This study was conducted by the Fukushima Regional Center as an adjunct study to the Japan Environment and Children’s Study. The study comprised 12 patients with early-onset HDP and 12 control subjects with healthy pregnancy whose medical background information was matched with that of the patients by propensity-score matching. Capillary electrophoresis and mass spectrometry-based quantitative analysis of charged metabolites were performed with the first-trimester maternal serum samples. Welch’s *t*-test was used to analyse metabolite peak areas in the two groups. A total of 166 charged metabolites were identified. The peak area of N-dimethylglycine and S-methylcysteine was significantly higher in the first-trimester serum of patients with early-onset HDP than in the controls. Conversely, the peak area of munic acid was significantly decreased in the serum of patients with early-onset HDP. Although we identified potential biomarkers for the prediction and diagnosis of early-onset HDP, no clear marker was identified because of a low statistical power.

## Introduction

Hypertensive disorders of pregnancy (HDP) complicate approximately 2.5% of all pregnancies in Japan^1^. They cause the death of approximately 30,000 women per year, accounting for 14% of maternal deaths worldwide^2,3^. HDP are diagnosed as a chronic type (hypertension predating pregnancy or diagnosed before 20 weeks of pregnancy) or de novo [(either pre-eclampsia (PE) or gestational hypertension (GH)]^4^ disorders. PE and gestational GH are the main types of HDP, which are conventionally defined as new-onset hypertension after 20 weeks of gestation, with (PE) or without (GH) signs of dysfunction in organs such as the kidney, liver, and placenta. HDP can also be categorised into early onset (Eo; onset before 34 weeks) and late onset (Lo; onset after 34 weeks) types. Eo-HDP is considered to be caused by the failure of the trophoblasts to invade the maternal spiral artery, which results in a high maternal vascular resistance^5^.

Among HDP, Eo-HDP occurs before 30 weeks in 0.3% of patients^1^, and it is associated with significant foetal mortality; therefore, the identification and treatment of women at a high risk of its development are a major challenge in modern obstetrics. Angiogenic factors, such as placental growth factor, vascular endothelial growth factor, soluble fms-related receptor tyrosine kinase 1, and endoglin^6^ are potential biomarkers for Eo-HDP. Although there are changes in the levels of these cytokines before the onset of clinical HDP symptoms, it is not clear whether these markers are effective for detecting Eo-HDP, as these changes occur relatively late in gestation. Therefore, the identification of early biomarkers for Eo-HDP would help identify women at the risk of Eo-HDP.

One approach to identify disease biomarkers is to use information-rich analytical tools, such as omic-scale biological methods, to characterise the composition of the target tissue in healthy individuals and patients. Metabolites are small molecule products of metabolism, and their chemical interactions in the body are necessary for life^7^. Metabolomic analysis involves the use of high-throughput analytical methods to identify and quantify metabolites, allowing the observation of dynamic changes in phenotype and system homeostasis. Recently, significant advances have been made in both metabolomic identification techniques^8,9^ and computational tools to analyse a large volume of data generated by metabolomic studies^10^. With this increased accessibility to vital data, metabolomics is expected to aid in the early diagnosis of a variety of complex diseases^11^. In this study, we conducted a comprehensive metabolome analysis of maternal serum collected during the first trimester, in order to identify biomarkers for Eo-HDP.

## Results

### Maternal background characteristics and obstetric outcomes in healthy and Eo-HDP-affected pregnancies

To identify early biomarkers of Eo-HDP, we analysed the first-trimester maternal serum of women who had healthy pregnancy and those with Eo-HDP. Table 1 shows the maternal backgrounds and obstetric outcomes of the two groups. As the controls were selected by propensity matching, there were no significant differences with regard to the maternal background. None of the women were smokers. Regarding obstetrics outcomes, the median gestational age at delivery was significantly different between the two groups (*p* < 0.01). The birth weight and placental weight were also significantly different between the groups (both *p* < 0.01); however, these factors are typically affected by the gestational age at delivery. The mean standard deviation (SD) of birth weight and percentage of babies that were small for their gestational age, which are not affected by the gestational age at delivery, was also significantly different between the two groups (both *p* < 0.01).

**Table 1:** Basic characteristics and obstetrics outcomes in the two groups.

	Eo-HDP	Control	*p *value
	(n = 12)	(n = 12)	
**Maternal background**			
Gestational age at examination (weeks)	12.0 (11.0–12.0)	12.0 (11.3–12.0)	0.63
Maternal age (years)	33 (29–38)	36 (28–38)	0.87
Height before pregnancy (cm)	158 (153–163)	163 (157–165)	0.18
Weight before pregnancy (kg)	67.8 (59.8–74.8)	70.0 (60.8–81.5)	0.80
BMI before pregnancy (kg/cm^2^)	27.4 (24.1–31.6)	25.7 (23.3–28.3)	0.59
Nulliparous, n (%)	58.3 (7)	41.7 (5)	0.34
Systolic blood pressure in the first trimester (mmHg)	134 (115–136)	129 (118–136)	0.59
Diastolic blood pressure in the first trimester (mmHg)	80 (71–94)	82 (77–91)	1.00
HbA1c in the first trimester (mg/dL)	5.4 (5.1–5.7)	5.3 (5.2–5.4)	0.38
**Obstetrics outcomes**			
Gestational age at delivery (weeks)	36 (30–37)	38 (38–39)	< 0.01
Birth weight (g)	1791 (1016–2627)	3213 (3081–3426)	< 0.01
SD of birth weight	− 1.8 (− 2.5 to − 0.27)	0.8 (0.2–1.4)	< 0.01
SGA, n (%)	6 (50)	0 (0)	< 0.05
Weight of placenta (g)	380 (260–527)	665 (564–713)	< 0.01

All continuous values represent the median (interquartile range) unless otherwise indicated.

*Eo* early onset, *HDP* hypertension disorders of pregnancy, *SD* standard deviation, *BMI* body mass index, *SGA* small for gestational age.

### Metabolomic analysis of the first-trimester maternal serum from women with healthy and Eo-HDP-affected pregnancies

**Figure 1:**
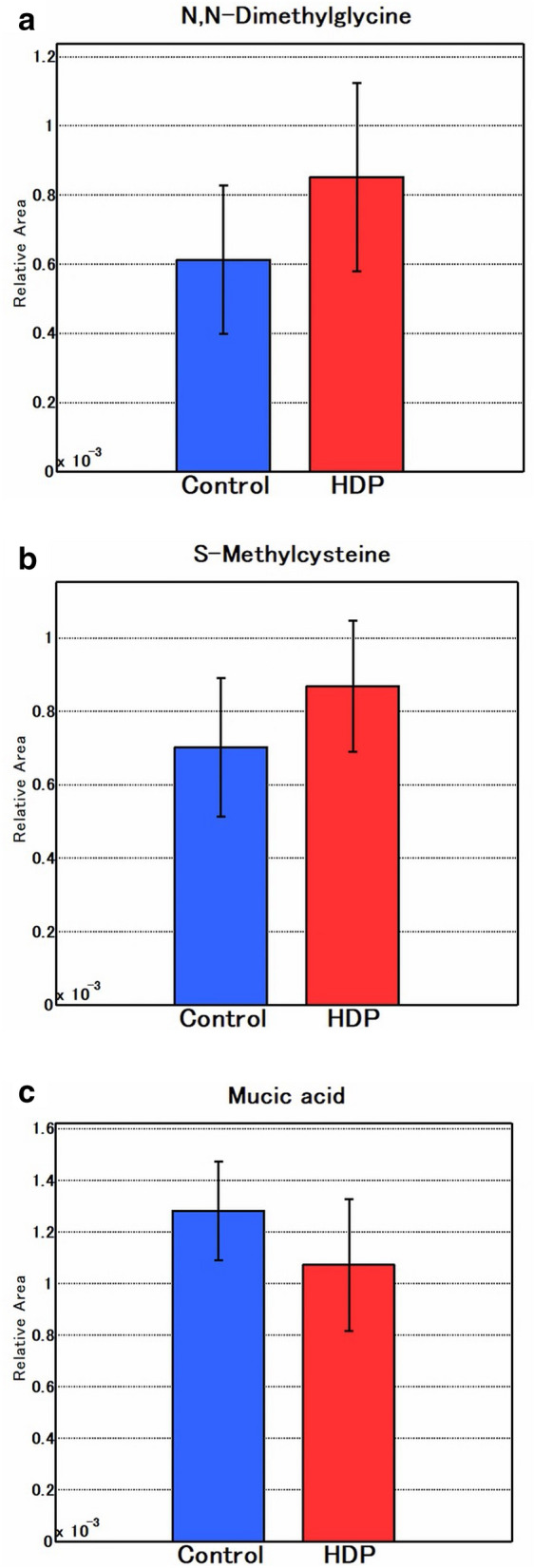
Three metabolites that were significantly different between the control and Eo-HDP samples. (**a**) N,N-Dimethylglycine, (**b**) S-methylcysteine, (**c**) munic acid.

A total of 166 charged metabolites were identified (Supplemental Table S1). Among them, the peak area of N-dimethylglycine and S-methylcysteine (SMC) was significantly higher in the first-trimester serum of patients with Eo-HDP than in the healthy controls (*p* = 0.026 and *p* = 0.037, respectively). Conversely, the peak area of munic acid was significantly decreased in the serum of patients with Eo-HDP (*p* = 0.033; Fig. 1).

**Figure 2:**
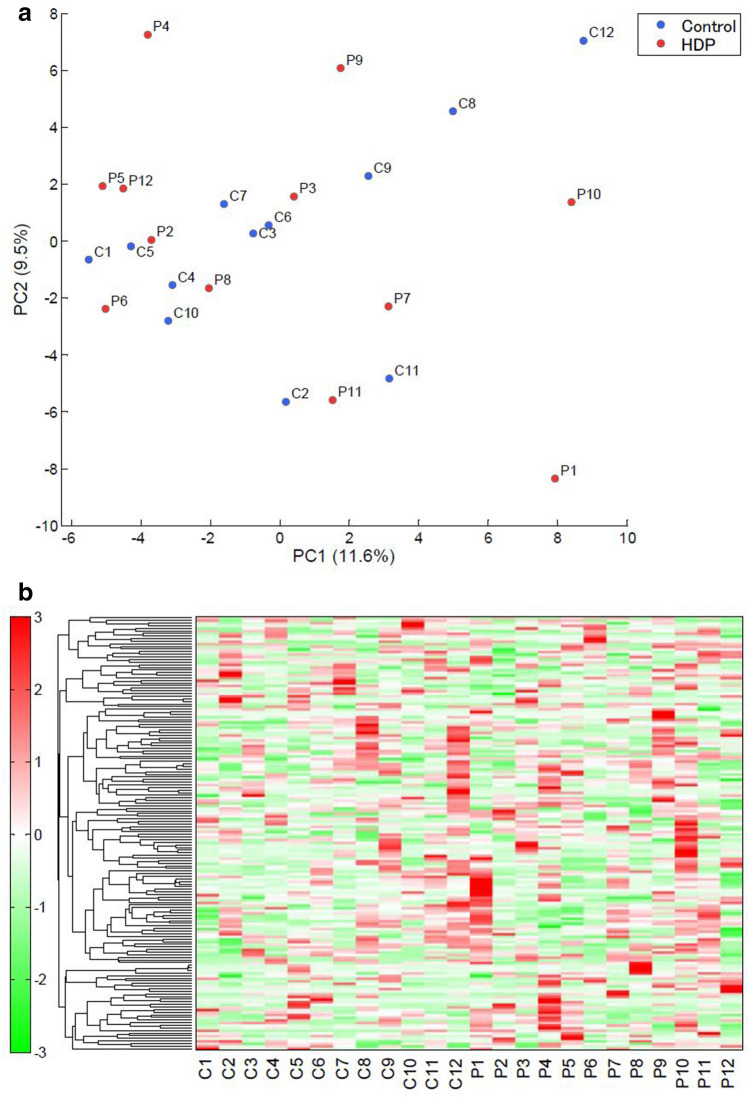
First trimester maternal serum metabolomic profiles of Eo-HDP-affected and healthy pregnancies. (**a**) PCA. Plots show the PCA scores obtained from maternal serum samples. Metabolically similar samples are located close to one other in the score plot. The x-axis indicates the main PC and the y-axis indicates the secondary PC. Each PC represents an axis in multidimensional space and corresponds to the direction of maximum variation in the original data. Numbers in parentheses indicate the contribution rates of each PC. (**b**): HCA. Rows and columns indicate metabolites and cases, respectively. Relative metabolite levels are represented in different colours, with red and green indicating increased and decreased levels, respectively.

We performed the principal component analysis (PCA) and hierarchical cluster analysis (HCA) of the data (Fig. 2a and b, respectively). The PCA did not clearly separate the patients with Eo-HDP from the healthy controls. The accumulated variance of PC1/PC2 was low, at 21.1%. The metabolome data were hierarchically clustered by both metabolites and sample groups to generate a heat map. However, the metabolite profiles of patients with Eo-HDP and healthy women were not readily distinguishable.

## Discussion

The major advantages of comprehensive metabolome analysis include its extremely high resolution and ability to simultaneously quantify charged low molecular weight compounds^12^. In this study, we conducted a comprehensive metabolomic analysis of the first-trimester serum of patients with Eo-HDP who were background-matched with healthy obstetrics individuals. Although we did not observe apparent changes in the metabolite patterns between Eo-HDP-affected and healthy pregnancies by PCA and HCA, there were significant changes in specific metabolites between the groups, including N-dimethylglycine, SMC, and munic acid. To the best of our knowledge, this is the first study to report these metabolites as potential early biomarkers for Eo-HDP.

Another advantage of metabolomic analysis is its hypothesis-generating ability. An important practical application of metabolomic analysis is to identify biomarkers for disorders that are complex and difficult to diagnose, such as ovarian cancer^13^ and schizophrenia^14^. In obstetrics, metabolite analysis of the first trimester maternal plasma or serum could be routinely used to detect early biomarkers for the prediction of new-onset hypertension during pregnancy. Accordingly, several studies have attempted to identify early biomarkers to predict PE and/or GH^15–17^ and several metabolites have been identified as candidates. Unlike previous studies, here, we retrospectively selected first trimester control samples whose basic characteristics were matched with those of samples from patients who later developed Eo-HDP, allowing the identification of new potential biomarkers for the disease.

N-Dimethylglycine is an intermediate metabolite in the biosynthesis of glycine from choline. The reason why the concentration of N-dimethylglycine increases during the first trimester among women with Eo-HDP is speculative. A previous metabolomic analysis of human amniotic fluid among healthy pregnant individuals revealed that both choline and N-dimethylglycine increased in the second and third trimesters^18^. Although, in the present study, we did not find a significant difference in choline concentration, there might be an association between choline and N-dimethylglycine, which is an intermediate metabolite of choline metabolism. Choline is an essential nutrient for cellular structural integrity and signal transduction, and is a precursor of the neurotransmitter acetylcholine^19^. In a cohort study, Roe et al. reported that a higher plasma concentration of choline was associated with unfavourable cardiometabolic risk factors, such as a lower concentration of high-density lipoprotein cholesterol, higher concentration of total homocysteine, higher body mass index, and greater odds of large vessel cerebrovascular disease or history of cardiovascular disease^20^.

This study is the first to report changes in SMC and munic acid levels in biological samples from pregnant women. SMC is a hydrophilic cysteine-containing compound naturally synthesised by several *Allium* species, such as garlic and onion^21^. It has been reported that garlic extract protects the lungs and bronchial smooth muscle cell lines by increasing the intracellular glutathione content^22^. Dietary SMC intake exhibited anti-oxidative and anti-inflammatory effects against ethanol-induced liver injury in mice^23^. On the contrary, another study reported that high doses of SMC increased the concentration of the oxidative stress marker nitric oxide and activity of superoxide dismutase in rat hearts^24^. Therefore, excessive SMC can cause cardiac oxidative stress-induced damage and subsequent cardiotoxicity. This suggests that the increased SMC concentration observed in the first-trimester maternal serum of patients with Eo-HDP may induce oxidative stress and endothelial dysfunction, inducing the pathogenesis of new-onset hypertension. Munic acid is a dicarboxylic sugar acid that results from the oxidation of galactose with dilute nitric acid. It is used in skin care products, and its monomers can be chemically converted for polymer production^25^. However, the biological function of munic acid is not known. Other than the three metabolites, we did not find changes in the levels of other markers in the same pathway. Therefore, the changes in these metabolites may have been caused by their deprivation from dietary intake, rather than the parent molecules.

A strength of this study is the use of data from the first large-scale birth cohort study conducted by the Japanese Government, the Japan Environmental Children's Study (JECS), which was performed with meticulous attention to data collection. We prospectively collected first-trimester maternal serum from women whose obstetrics outcomes were then unknown, as an adjunct to the JECS. Nevertheless, this study also has potential limitations. First, as we performed the *t*-test for 166 metabolites simultaneously, statistical type 1 errors may exist. Although we used the propensity score to match the maternal basic background, the present study was conducted with a small sample size and high significance threshold (*p* < 0.05). Furthermore, the PCA plot explained only 21% of the effect, reflecting the imbalance between samples and markers; we cannot deny the possibility of finding three metabolites by chance result. Second, although obstetric outcomes were based on medical records, in this study, we focused on HDP, which does not distinguish GH from PE. As PE is accompanied by proteinuria and other organ dysfunction, its complications affect the mother and baby more severely than GH^4^. However, GH and PE may also be undistinguishable in terms of long-term cardiovascular risk, including the risk of chronic hypertension^26^. Third, because we chose control patients based on the maternal background of the patients with Eo-HDP, they may not represent truly healthy maternity. For example, the median maternal age of both Eo-HDP (36 years) and control (33 years) groups was high compared to vital statistics acquired during the same period^27^.

HDP remain a major cause of maternal mortality and morbidity worldwide, and their pathogeneses are not well understood. As metabolites form the building blocks of genes, transcripts, and proteins, the metabolome has the potential to provide more fundamental and global information than other—omic strategies. Metabolomic data can help identify new biomarkers and expand our understanding of disease mechanisms. Although we identified potential biomarkers for the prediction and diagnosis of Eo-HDP, no clear marker was identified because of the low statistical power. Further studies with a large number of participants and strong statistical power are required.

## Materials and methods

### Study design

In this study, we used data and samples from the JECS, a Japanese Government-funded birth cohort study started in January 2011 to investigate the effects of several environmental factors on children's health^28^. The present study was conducted as an adjunct study to the JECS.

This study was reviewed and approved by the Ministry of the Environment’s Institutional Review Board on Epidemiological Studies (No. 1305151) and by the Ethics Committee of Fukushima Medical University (No. 1603). All methods were conducted in accordance with the Helsinki Declaration and other nationally valid regulations and guidelines.

### Samples

Serum samples were prospectively collected from pregnant women who provided written informed consent at approximately 12 weeks of gestation in Fukushima Regional Center. Women with complications before pregnancy and insufficient data were excluded from the present study. We could obtain 5070 maternal serum samples, and 22 patients (0.4%) developed Eo-HDP. Among them, 9 patients (4 with glucose intolerance during pregnancy, 3 with maternal renal disease before pregnancy, and 1 with maternal autoimmune disease) and 1 patient with insufficient data were excluded. In a retrospective manner, 12 samples from patients who subsequently developed Eo-HDP were matched with 12 samples from women with healthy pregnancy. The basic characteristics of patients and controls were matched using 1:1 propensity score matching and then caliper matching. Serum samples were stored at − 80 °C for metabolome analysis.

### Outcomes and basic characteristics

Obstetric outcomes included the presence of Eo-HDP, mode of delivery, gestational age, and birth weight, and the SD of birth weight accounting for gestational age. HDP was defined as new-onset hypertension (≥ 140/90 mmHg) after conception. In the present study, Eo-HDP was defined as HDP onset before 30 weeks of gestation. The SD was calculated according to Japanese neonatal anthropometric charts^29^, which accounted for gestational age, sex, and parity. Babies with values < − 1.5 SDs were defined as small for their gestational age^30^. Placental weight was measured by a midwife soon after delivery.

The following items were considered basic maternal characteristics: height and weight before pregnancy, age at delivery, number of previous deliveries, method of conception, smoking status, and systolic and diastolic blood pressure at 12 weeks of gestation. The method of conception was categorised as natural conception or conception after infertility treatment.

### Metabolomic analysis

Metabolome analysis was performed with serum samples according to the protocol provided by Human Metabolome Technologies, Inc. (HMT, Yamagata, Japan). Serum (50 µL) was combined with methanol containing internal standards (450 µL; Solution ID: H3304-1002) at 0 °C for enzyme inactivation. The extract was thoroughly mixed with chloroform (500 µL) and Milli-Q water (200 µL) and centrifuged at 2300 × *g* for 5 min at 4 °C. The upper aqueous layer (400 µL) was filtered by centrifugation using a Millipore 5-kDa cutoff filter to remove proteins. The filtrate was concentrated by centrifugation and resuspended in Milli-Q water (50 µL) for capillary electrophoresis (CE)–mass spectrometry (MS) analysis. Metabolome analysis was performed by Human Metabolome Technologies Inc.

CE-time of flight (TOF)-MS was performed on an Agilent CE System equipped with a 6210 TOF–MS and 1100 isocratic high-performance liquid chromatography pump, using the G1603A CE-MS adapter kit and G1607A CE-electrospray ionisation-MS sprayer kit (all Agilent Technologies, Waldbronn, Germany). The systems were controlled by Agilent G2201AA ChemStation software version B.03.01 for CE. The metabolites were resolved on a fused silica capillary (50 μm *i.d.* × 80 cm total length), with commercial electrophoresis buffers (Solutions H3301-1001 and H3302-1021 for cation and anion analyses, respectively; Human Metabolome Technologies) as the electrolytes. Cation analysis samples (approximately 10 nL) were injected at a pressure of 50 mbar for 10 s; anion samples (approximately 25 nL) were injected for 25 s. The spectrometer scanned from 50 to 1000 m*/z*. Other conditions were as previously described^8,9,31^.

Peaks were extracted using the automatic integration software MasterHands (Keio University, Tsuruoka, Japan) to obtain peak information, including the *m/z*, migration time (MT), and peak area^32^. Signal peaks corresponding to isotopomers, adduct ions, and other product ions of known metabolites were excluded, and the remaining peaks were annotated with putative metabolites from the Human Metabolome Technologies metabolite database based on their MTs and *m/z* values. The tolerance value for peak annotation was ± 0.5 min for MT and ± 10 ppm for *m/z*. Peak areas were normalised against those of the internal standards, and the resultant relative area values were further normalised by sample amount.

### Statistical analysis

For maternal background analysis, Mann–Whitney* U* test and *Fisher exact* test were used for continuous and categorical variables, respectively. Welch’s *t*-test was used to compare metabolite concentrations. Hierarchical cluster analysis (HCA) and principal component analysis (PCA) were performed using our proprietary software PeakStat and SampleStat, respectively. Detected metabolites were plotted on metabolic pathway maps using Visualization and Analysis of Networks containing Experimental Data (VANTED) software^9^. SPSS version 24 (IBM Corp., Armonk, NY, USA) was used for statistical analysis. In the metabolome analysis, SampleStat version 3.14 was used for statistical analyses. The level of statistical significance was set at *p* < 0.05.

## Supplementary information

Supplementary Table.

## Data Availability

The data that support the findings of this study are available from the corresponding author, H.K., upon reasonable request.
