# Supplementary material for: Comprehensive metabolomic analysis of first-trimester serum identifies biomarkers of early-onset hypertensive disorder of pregnancy
Source: Sci Rep. 2020 Aug 17;10:13857. doi: 10.1038/s41598-020-70974-3 (PMC7431422; doi:10.1038/s41598-020-70974-3)
Supplement: Supplementary file 1 — Supplementary Table. [file 41598_2020_70974_MOESM1_ESM.docx]

Title: Comprehensive metabolic analysis of first-trimester serum identifies biomarkers of early-onset hypertensive disorder of pregnancy

Hyo Kyozuka^1,2^, Toma Fukuda^1,2^, Tsuyoshi Murata^1,2^, Yuta Endo^1^, Aya Kanno^1^, Shun Yasuda^1^, Akiko Yamaguchi^1,2^, Miho Ono^1^, Akiko Sato^2^, Koichi Hashimoto^2^, Keiya Fujimori^1,2^

Supplemental Table S1.

Metabolite changes between control and Eo-HDP samples

| Compound | Control | | Eo-HDP | | Eo-HDP vs Control | |
| --- | --- | --- | --- | --- | --- | --- |
|  | Mean | SD | Mean | SD | Ratio | *p*-value* |
| 1-Methyl-4-imidazoleacetic acid | 8.9E-05 | 1.3E-05 | 9.4E-05 | 1.9E-05 | 1.1 | 0.516 |
| 1-Methyladenosine | 4.3E-05 | 1.2E-05 | 4.3E-05 | 1.3E-05 | 1.0 | 0.983 |
| 1-Methylhistidine 3-Methylhistidine | 1.1E-03 | 5.2E-04 | 1.1E-03 | 6.0E-04 | 1.0 | 0.815 |
| 1-Methylnicotinamide | 1.0E-04 | 3.5E-05 | 8.7E-05 | 3.3E-05 | 0.8 | 0.304 |
| 10-Hydroxydecanoic acid | 7.4E-05 | N.A. | 8.6E-05 | 3.2E-05 | 1.2 | N.A. |
| 2-Aminoisobutyric acid 2-Aminobutyric acid | 4.2E-03 | 8.9E-04 | 4.4E-03 | 1.4E-03 | 1.1 | 0.631 |
| 2-Hydroxy-4-methylvaleric acid | 1.2E-04 | 1.2E-05 | 1.1E-04 | 2.8E-05 | 1.0 | 0.943 |
| 2-Hydroxybutyric acid | 2.4E-03 | 9.6E-04 | 2.1E-03 | 9.8E-04 | 0.9 | 0.438 |
| 2-Hydroxyglutaric acid | 2.3E-04 | 9.5E-05 | 2.0E-04 | 8.4E-05 | 0.9 | 0.438 |
| 2-Hydroxyisobutyric acid | 1.1E-04 | 2.2E-05 | 1.1E-04 | 2.4E-05 | 1.0 | 0.817 |
| 2-Hydroxyvaleric acid | 7.6E-04 | 1.7E-04 | 7.5E-04 | 2.3E-04 | 1.0 | 0.973 |
| 2-Oxoglutaric acid | 1.0E-03 | 1.5E-04 | 1.1E-03 | 6.1E-04 | 1.1 | 0.895 |
| 2-Oxoisovaleric acid | 6.8E-04 | 1.9E-04 | 7.1E-04 | 2.5E-04 | 1.0 | 0.721 |
| 3-Aminobutyric acid | 3.8E-04 | 2.1E-04 | 5.8E-04 | 3.3E-04 | 1.5 | 0.115 |
| 3-Hydroxybutyric acid | 2.6E-03 | 2.2E-03 | 3.9E-03 | 6.4E-03 | 1.5 | 0.502 |
| 3-Indoxylsulfuric acid | 4.0E-04 | 7.5E-05 | 3.6E-04 | 1.4E-04 | 0.9 | 0.327 |
| 3-Phenylpropionic acid | 2.3E-04 | 1.0E-04 | 2.0E-04 | 4.8E-05 | 0.9 | 0.352 |
| 4-Guanidinobutyric acid | N.A. | N.A. | 8.5E-05 | N.A. | 1< | N.A. |
| 4-Methyl-2-oxovaleric acid 3-Methyl-2-oxovaleric acid | 4.6E-03 | 1.3E-03 | 4.2E-03 | 1.5E-03 | 0.9 | 0.576 |
| 5-Hydroxylysine | 7.8E-05 | 2.0E-05 | 7.0E-05 | 1.5E-05 | 0.9 | 0.333 |
| 5-Methylcytosine | N.A. | N.A. | 3.4E-05 | 1.2E-05 | 1< | N.A. |
| 5-Oxoproline | 6.9E-03 | 1.4E-03 | 6.5E-03 | 9.4E-04 | 0.9 | 0.374 |
| 8-Hydroxyoctanoic acid 2-Hydroxyoctanoic acid | 6.0E-05 | N.A. | 5.3E-05 | 9.6E-06 | 0.9 | N.A. |
| ADMA | 2.2E-04 | 2.0E-05 | 2.1E-04 | 3.2E-05 | 1.0 | 0.839 |
| Ala | 1.0E-01 | 1.3E-02 | 9.9E-02 | 9.2E-03 | 1.0 | 0.630 |
| *allo*-Threonine | 4.2E-04 | 1.6E-04 | 4.2E-04 | 1.5E-04 | 1.0 | 0.944 |
| Arg | 5.5E-02 | 1.1E-02 | 6.1E-02 | 1.5E-02 | 1.1 | 0.275 |
| Argininosuccinic acid | 4.0E-05 | 1.0E-05 | 4.3E-05 | 1.5E-05 | 1.1 | 0.675 |
| Ascorbate 2-sulfate | 3.1E-04 | 6.2E-05 | 2.9E-04 | 6.4E-05 | 0.9 | 0.381 |
| Asn | 1.4E-02 | 2.2E-03 | 1.5E-02 | 3.2E-03 | 1.1 | 0.460 |
| Asp | 2.1E-02 | 2.7E-03 | 2.1E-02 | 5.3E-03 | 1.0 | 0.989 |
| Betaine | 9.1E-03 | 1.6E-03 | 1.2E-02 | 4.8E-03 | 1.3 | 0.067 |
| Betonicine | 4.0E-04 | 4.9E-04 | 6.5E-04 | 8.5E-04 | 1.6 | 0.701 |
| Butyrylcarnitine | 1.1E-04 | 2.0E-05 | 1.5E-04 | 6.8E-05 | 1.4 | 0.120 |
| Caffeine | 6.8E-04 | 4.1E-04 | 3.8E-04 | 2.1E-05 | 0.6 | 0.490 |
| Carnitine | 1.7E-02 | 1.8E-03 | 1.9E-02 | 3.3E-03 | 1.1 | 0.105 |
| Cholic acid | N.A. | N.A. | 2.3E-04 | 2.5E-04 | 1< | N.A. |
| Choline | 1.1E-01 | 2.3E-02 | 9.3E-02 | 3.2E-02 | 0.9 | 0.222 |
| *cis*-Aconitic acid | 4.6E-04 | 8.9E-05 | 4.4E-04 | 9.1E-05 | 1.0 | 0.538 |
| Citric acid | 1.1E-02 | 2.2E-03 | 1.1E-02 | 2.2E-03 | 1.0 | 0.903 |
| Citrulline | 4.6E-03 | 1.3E-03 | 4.3E-03 | 1.2E-03 | 0.9 | 0.596 |
| Creatine | 1.4E-02 | 3.1E-03 | 1.5E-02 | 5.0E-03 | 1.0 | 0.787 |
| Creatinine | 1.2E-02 | 1.6E-03 | 1.2E-02 | 2.1E-03 | 1.0 | 0.984 |
| Cystine | 8.8E-04 | 2.2E-04 | 8.0E-04 | 2.5E-04 | 0.9 | 0.441 |
| Decanoic acid | 2.2E-04 | 5.9E-05 | 3.5E-04 | 1.8E-04 | 1.6 | 0.055 |
| Diethanolamine | 6.4E-04 | 4.8E-04 | 4.2E-04 | 2.6E-04 | 0.7 | 0.182 |
| Dyphylline | 1.3E-02 | 2.4E-03 | 1.2E-02 | 2.5E-03 | 1.0 | 0.535 |
| Ergothioneine | 3.9E-04 | N.A. | 1.1E-03 | 1.1E-03 | 2.8 | N.A. |
| Ethanolamine | 2.3E-03 | 3.3E-04 | 2.2E-03 | 2.3E-04 | 1.0 | 0.741 |
| Formylanthranilic acid | 7.5E-05 | 6.0E-06 | 1.0E-04 | 1.9E-05 | 1.3 | 0.083 |
| Fumaric acid monomethyl ester | 1.2E-04 | 2.9E-05 | 1.4E-04 | 4.3E-05 | 1.2 | 0.264 |
| Galactosamine Glucosamine | 1.5E-04 | 7.2E-05 | 1.7E-04 | 1.2E-04 | 1.1 | 0.661 |
| Gln | 8.4E-02 | 1.0E-02 | 8.9E-02 | 1.7E-02 | 1.1 | 0.421 |
| Glu | 5.9E-02 | 1.2E-02 | 5.8E-02 | 1.3E-02 | 1.0 | 0.848 |
| Glu-Glu | 9.0E-05 | 3.0E-05 | 9.7E-05 | 5.0E-05 | 1.1 | 0.679 |
| Gluconic acid | 4.0E-04 | 9.7E-05 | 4.0E-04 | 7.2E-05 | 1.0 | 0.992 |
| Gluconolactone | 2.5E-04 | 6.1E-05 | 2.4E-04 | 5.0E-05 | 1.0 | 0.717 |
| Glucuronic acid Galacturonic acid | 1.5E-04 | 2.4E-05 | 1.5E-04 | 2.5E-05 | 1.0 | 0.807 |
| Gly | 4.7E-02 | 5.7E-03 | 4.7E-02 | 3.9E-03 | 1.0 | 0.716 |
| Gly-Asp | 2.1E-04 | 7.3E-05 | 1.8E-04 | 4.0E-05 | 0.8 | 0.194 |
| Gly-Gly | 4.4E-04 | 2.7E-04 | 4.3E-04 | 1.2E-04 | 1.0 | 0.930 |
| Glyceric acid | 2.0E-03 | 4.0E-04 | 2.0E-03 | 3.9E-04 | 1.0 | 0.643 |
| Glycerol | 1.3E-01 | 2.0E-02 | 1.4E-01 | 2.0E-02 | 1.0 | 0.488 |
| Glycerol 2-phosphate | N.A. | N.A. | 8.4E-05 | 1.9E-05 | 1< | N.A. |
| Glycerol 3-phosphate | 1.3E-03 | 6.0E-04 | 1.1E-03 | 4.9E-04 | 0.9 | 0.403 |
| Glycerophosphocholine | 1.0E-02 | 5.0E-03 | 7.5E-03 | 4.9E-03 | 0.7 | 0.192 |
| Glycocholic acid | 1.0E-04 | 2.1E-05 | 1.3E-04 | 6.2E-05 | 1.3 | 0.287 |
| Guanidinosuccinic acid | 5.6E-05 | 1.4E-05 | 4.2E-05 | 1.4E-05 | 0.7 | 0.228 |
| Guanidoacetic acid | 5.5E-04 | 9.1E-05 | 5.6E-04 | 1.0E-04 | 1.0 | 0.788 |
| Guanosine | 4.6E-05 | 8.4E-06 | 5.6E-05 | 2.7E-05 | 1.2 | 0.699 |
| Hexanoic acid | 1.2E-04 | 1.4E-05 | 1.1E-04 | 2.1E-05 | 1.0 | 0.655 |
| Hippuric acid | 2.1E-04 | 1.1E-04 | 1.5E-04 | 7.4E-05 | 0.7 | 0.240 |
| His | 3.7E-02 | 4.7E-03 | 3.8E-02 | 6.4E-03 | 1.0 | 0.758 |
| His-Glu | 1.0E-04 | 3.4E-05 | 1.2E-04 | 2.8E-05 | 1.2 | 0.254 |
| Homocitrulline | 1.3E-04 | 3.0E-05 | 1.0E-04 | 2.7E-05 | 0.8 | 0.322 |
| Homovanillic acid | 7.4E-05 | 1.0E-05 | 7.9E-05 | 1.4E-05 | 1.1 | 0.454 |
| Hydroxyproline | 2.0E-03 | 6.6E-04 | 1.8E-03 | 3.1E-04 | 0.9 | 0.316 |
| Hypotaurine | 6.1E-04 | 2.0E-04 | 6.6E-04 | 1.8E-04 | 1.1 | 0.501 |
| Hypoxanthine | 2.2E-02 | 8.9E-03 | 2.3E-02 | 8.5E-03 | 1.0 | 0.778 |
| Ile | 5.0E-02 | 6.0E-03 | 4.8E-02 | 1.3E-02 | 1.0 | 0.576 |
| Imidazolelactic acid | 8.9E-05 | 1.8E-05 | 9.0E-05 | 2.1E-05 | 1.0 | 0.881 |
| Indole-3-acetic acid | 1.1E-04 | 4.5E-05 | 1.0E-04 | 2.7E-05 | 0.9 | 0.567 |
| Inosine | 1.6E-03 | 2.0E-03 | 2.5E-03 | 2.1E-03 | 1.6 | 0.303 |
| Isethionic acid | 9.5E-05 | 1.6E-05 | 1.0E-04 | 2.4E-05 | 1.1 | 0.311 |
| Isobutyric acid Butyric acid | 1.4E-04 | 4.0E-05 | 1.6E-04 | 3.0E-05 | 1.2 | 0.582 |
| Isocitric acid | 5.6E-04 | 9.9E-05 | 5.3E-04 | 1.0E-04 | 1.0 | 0.583 |
| Isovaleric acid Valeric acid | 2.8E-04 | 8.2E-05 | 3.1E-04 | 9.2E-05 | 1.1 | 0.619 |
| Isovalerylcarnitine | N.A. | N.A. | 1.3E-04 | 1.6E-05 | 1< | N.A. |
| Kynurenine | 4.1E-04 | 1.0E-04 | 4.3E-04 | 7.1E-05 | 1.0 | 0.578 |
| Lactic acid | 3.8E-01 | 7.6E-02 | 3.5E-01 | 7.3E-02 | 0.9 | 0.445 |
| Lauric acid | 1.1E-03 | 2.3E-04 | 1.1E-03 | 2.7E-04 | 1.0 | 0.733 |
| Leu | 1.2E-01 | 1.2E-02 | 1.2E-01 | 3.0E-02 | 1.0 | 0.929 |
| Lys | 5.6E-02 | 7.8E-03 | 5.5E-02 | 1.1E-02 | 1.0 | 0.937 |
| Malic acid | 1.2E-03 | 3.0E-04 | 1.1E-03 | 2.3E-04 | 0.9 | 0.581 |
| Met | 8.0E-03 | 1.9E-03 | 7.2E-03 | 2.0E-03 | 0.9 | 0.317 |
| Methionine sulfoxide | 5.6E-04 | 1.6E-04 | 6.0E-04 | 1.7E-04 | 1.1 | 0.542 |
| Mevalonic acid | 8.3E-05 | 2.8E-05 | 1.0E-04 | 1.9E-05 | 1.2 | 0.171 |
| Mucic acid | 1.3E-03 | 1.9E-04 | 1.1E-03 | 2.6E-04 | 0.8 | 0.033 |
| Myristoleic acid | 1.6E-04 | 7.0E-05 | 1.3E-04 | 1.1E-04 | 0.8 | 0.499 |
| *N*,*N*-Dimethylglycine | 6.1E-04 | 2.1E-04 | 8.5E-04 | 2.7E-04 | 1.4 | 0.026 |
| *N*-Acetyl-β-alanine | 6.3E-05 | 1.6E-05 | 6.4E-05 | 1.5E-05 | 1.0 | 0.895 |
| *N*-Acetylalanine | 1.0E-04 | 1.6E-05 | 1.0E-04 | 1.7E-05 | 1.0 | 0.758 |
| N-Acetylgalactosamine-1 N-Acetylmannosamine-1 N-Acetylglucosamine-1 | 1.0E-03 | 2.6E-04 | 1.1E-03 | 3.4E-04 | 1.1 | 0.522 |
| N-Acetylgalactosamine-2 N-Acetylmannosamine-2 N-Acetylglucosamine-2 | 4.1E-04 | 7.9E-05 | 4.0E-04 | 4.6E-05 | 1.0 | 0.767 |
| *N*-Acetylglycine | 9.5E-05 | 4.2E-05 | 1.3E-04 | 9.8E-05 | 1.3 | 0.397 |
| *N*-Acetyllysine | 2.1E-04 | 1.1E-04 | 1.9E-04 | 5.2E-05 | 0.9 | 0.662 |
| *N*-Acetylornithine | 4.3E-04 | 1.1E-04 | 5.3E-04 | 1.7E-04 | 1.2 | 0.110 |
| *N*-Acetylputrescine | 5.2E-05 | 9.2E-06 | 5.0E-05 | 6.8E-06 | 1.0 | 0.754 |
| *N*-Acetylserine | 1.3E-04 | 9.0E-06 | 1.4E-04 | 1.2E-05 | 1.1 | 0.271 |
| *N*-Ethylglycine | 1.7E-04 | 4.0E-05 | 1.1E-04 | 4.0E-06 | 0.6 | 0.281 |
| *N*-Methylproline | 4.5E-04 | 4.6E-04 | 1.1E-03 | 1.6E-03 | 2.5 | 0.367 |
| *N*^2^-Phenylacetylglutamine | 1.4E-04 | 6.2E-05 | 1.4E-04 | 6.4E-05 | 1.0 | 0.897 |
| *N*^5^-Ethylglutamine | 3.0E-04 | N.A. | 3.6E-04 | N.A. | 1.2 | N.A. |
| *N*^6^-Acetyllysine | 1.3E-04 | 6.4E-06 | 1.1E-04 | 2.0E-05 | 0.9 | 0.114 |
| Nicotinamide | 1.1E-04 | 1.4E-05 | 1.2E-04 | 7.8E-06 | 1.1 | 0.196 |
| *O*-Acetylcarnitine | 5.6E-03 | 1.6E-03 | 6.1E-03 | 3.0E-03 | 1.1 | 0.588 |
| *O*-Acetylhomoserine 2-Aminoadipic acid | 1.7E-04 | 3.6E-05 | 1.7E-04 | 5.6E-05 | 1.0 | 0.801 |
| *o*-Hydroxybenzoic acid | 4.7E-03 | 4.1E-03 | 2.4E-03 | 2.0E-03 | 0.5 | 0.575 |
| *o*-Hydroxyhippuric acid | 1.5E-04 | 4.8E-05 | 1.6E-04 | N.A. | 1.1 | N.A. |
| Octanoic acid | 1.8E-04 | 4.0E-05 | 3.0E-04 | 1.9E-04 | 1.7 | 0.068 |
| Octanoylcarnitine | 1.3E-04 | 7.3E-05 | 1.4E-04 | 7.1E-05 | 1.1 | 0.869 |
| Ornithine | 2.2E-02 | 3.4E-03 | 1.9E-02 | 4.0E-03 | 0.9 | 0.156 |
| *p*-Anisic acid *o*-Hydroxyphenylacetic acid Mandelic acid Phenoxyacetic acid | 2.3E-04 | N.A. | 9.9E-05 | 6.8E-06 | 0.4 | N.A. |
| Pelargonic acid | 2.1E-04 | 4.2E-05 | 2.1E-04 | 2.9E-05 | 1.0 | 0.980 |
| Phe | 7.5E-02 | 8.1E-03 | 7.6E-02 | 1.2E-02 | 1.0 | 0.883 |
| Pipecolic acid | 8.8E-04 | 2.3E-04 | 1.0E-03 | 7.1E-04 | 1.2 | 0.509 |
| Piperidine | 2.4E-04 | 9.0E-05 | 2.2E-04 | 1.2E-04 | 0.9 | 0.749 |
| Pro | 6.4E-02 | 7.9E-03 | 6.6E-02 | 1.3E-02 | 1.0 | 0.738 |
| Pyruvic acid | 1.1E-03 | N.A. | 1.8E-03 | 1.1E-03 | 1.7 | N.A. |
| Quinic acid | 2.6E-04 | 2.1E-04 | 1.5E-04 | 1.0E-04 | 0.6 | 0.491 |
| *S*-Methylcysteine | 7.0E-04 | 1.9E-04 | 8.7E-04 | 1.8E-04 | 1.2 | 0.037 |
| Sarcosine | 3.3E-04 | 9.5E-05 | 3.0E-04 | 1.1E-04 | 0.9 | 0.564 |
| SDMA | 2.1E-04 | 3.7E-05 | 2.1E-04 | 3.2E-05 | 1.0 | 0.859 |
| Ser | 5.2E-02 | 5.9E-03 | 5.1E-02 | 8.5E-03 | 1.0 | 0.658 |
| Ser-Glu | 1.4E-04 | 4.6E-05 | 1.4E-04 | 5.9E-05 | 1.0 | 0.998 |
| Serotonin | 1.2E-04 | 3.8E-05 | 9.9E-05 | 3.4E-05 | 0.8 | 0.154 |
| Stachydrine | 1.9E-03 | 2.5E-03 | 3.5E-03 | 6.3E-03 | 1.9 | 0.413 |
| Succinic acid | 5.3E-04 | 1.2E-04 | 5.0E-04 | 8.1E-05 | 1.0 | 0.578 |
| Sulfotyrosine | 6.2E-05 | 7.5E-06 | 6.1E-05 | 7.1E-06 | 1.0 | 0.867 |
| Taurine | 8.3E-03 | 1.2E-03 | 8.9E-03 | 1.8E-03 | 1.1 | 0.309 |
| Taurocholic acid | 1.1E-04 | 2.6E-05 | 8.7E-05 | 3.2E-05 | 0.8 | 0.419 |
| Terephthalic acid | 2.2E-04 | 1.2E-05 | 2.2E-04 | 1.2E-05 | 1.0 | 0.837 |
| Theobromine | 3.3E-04 | 1.1E-04 | 3.7E-04 | 1.9E-04 | 1.1 | 0.826 |
| Thr | 4.4E-02 | 9.1E-03 | 4.4E-02 | 1.2E-02 | 1.0 | 0.917 |
| Threonic acid | 1.4E-03 | 2.2E-04 | 1.4E-03 | 2.7E-04 | 1.0 | 0.929 |
| Triethanolamine | 6.8E-05 | 1.2E-05 | 6.6E-05 | 1.9E-05 | 1.0 | 0.798 |
| Trigonelline | 2.1E-04 | 1.5E-04 | 1.8E-04 | 2.3E-04 | 0.8 | 0.698 |
| Trimethylamine *N*-oxide | 1.2E-03 | 1.0E-03 | 9.6E-04 | 9.6E-04 | 0.8 | 0.579 |
| Trp | 2.1E-02 | 3.4E-03 | 2.1E-02 | 3.8E-03 | 1.0 | 0.755 |
| Tyr | 1.9E-02 | 2.2E-03 | 2.0E-02 | 5.5E-03 | 1.0 | 0.600 |
| Uracil | 1.4E-04 | 7.8E-06 | 1.4E-04 | 1.5E-05 | 1.0 | 0.756 |
| Urea | 2.2E-01 | 5.3E-02 | 2.2E-01 | 5.1E-02 | 1.0 | 0.829 |
| Uric acid | 1.7E-02 | 3.3E-03 | 1.6E-02 | 3.7E-03 | 1.0 | 0.910 |
| Uridine | 1.4E-03 | 2.4E-04 | 1.3E-03 | 2.4E-04 | 1.0 | 0.596 |
| Urocanic acid | 5.8E-05 | 1.0E-05 | 7.0E-05 | 1.9E-05 | 1.2 | 0.233 |
| Val | 1.1E-01 | 7.1E-03 | 1.2E-01 | 2.4E-02 | 1.1 | 0.310 |
| Xanthine | 9.3E-04 | 2.4E-04 | 9.6E-04 | 2.4E-04 | 1.0 | 0.765 |
| XC0016 | 8.1E-04 | 2.9E-04 | 8.8E-04 | 3.5E-04 | 1.1 | 0.593 |
| XC0040 | 3.2E-05 | 1.1E-05 | 5.5E-05 | 1.7E-05 | 1.7 | 0.268 |
| XC0061 | 2.2E-04 | 3.1E-05 | 2.5E-04 | 8.8E-05 | 1.1 | 0.499 |
| XC0065 | 3.9E-05 | 4.0E-06 | 4.4E-05 | 7.7E-06 | 1.1 | 0.141 |
| XC0120 | 1.4E-04 | 2.5E-05 | 1.4E-04 | 4.9E-05 | 0.9 | 0.594 |
| XC0126 | 8.6E-05 | 4.0E-05 | 7.1E-05 | 6.8E-06 | 0.8 | 0.573 |
| XC0132 | 3.4E-05 | 5.2E-06 | 3.3E-05 | 3.6E-06 | 1.0 | 0.803 |
| β-Ala | 5.5E-04 | 2.0E-04 | 5.7E-04 | 1.1E-04 | 1.0 | 0.737 |
| γ-Butyrobetaine | 6.4E-04 | 1.1E-04 | 6.5E-04 | 1.5E-04 | 1.0 | 0.879 |

Eo, early onset; HDP, hypertensive disorder of pregnancy; SD, standard deviation. N.A, not available

¶ The latter value was set as a denominator for the ratio of the averages between the two groups

**P* values were calculated by Welch’s *t*-test
